# Processed eggshell membrane powder regulates cellular functions and increase MMP-activity important in early wound healing processes

**DOI:** 10.1371/journal.pone.0201975

**Published:** 2018-08-06

**Authors:** Tram T. Vuong, Sissel B. Rønning, Tamer A. E. Ahmed, Kristiane Brathagen, Vibeke Høst, Maxwell T. Hincke, Henri-Pierre Suso, Mona E. Pedersen

**Affiliations:** 1 Nofima AS, Ås, Norway; 2 Department of Cellular and Molecular Medicine, Faculty of Medicine, University of Ottawa, Ottawa, Ontario, Canada; 3 Medical Biotechnology Department, Genetic Engineering and Biotechnology Research Institute, City of Scientific Research and Technology Applications (SRTA-City), Alexandria, Egypt; 4 Department of Innovation in Medical Education, Faculty of Medicine, University of Ottawa, Ottawa, Ontario, Canada; 5 Biovotec AS, Oslo, Norway; University of Portsmouth, UNITED KINGDOM

## Abstract

Avian eggshell membrane (ESM) is a natural biomaterial that has been used as an alternative natural bandage to cure wounds, and is available in large quantities from egg industries. We have previously demonstrated that processed eggshell membrane powder (PEP), aiming to be used in a low cost wound healing product, possesses anti-inflammatory properties. In this study, we further investigated effects of PEP on MMP activities *in vitro* (a dermal fibroblast cell culture system) and *in vivo* (a mouse skin wound healing model). Three days incubation with PEP in cell culture led to rearrangement of the actin-cytoskeleton and vinculin in focal adhesions and increased syndecan-4 shedding. In addition, we observed increased matrix metalloproteinase type 2 (MMP-2) enzyme activation, without effects on protein levels of MMP-2 or its regulators (membrane type 1 (MT1)-MMP and tissue inhibitor of matrix metalloproteinase type 2 (TIMP-2). Longer incubation (10 days) led to increased protein levels of MMP-2 and its regulators. We also observed an increased alpha-smooth muscle actin (α-SMA) production, suggesting an effect of PEP on myofibroblast differentiation. *In vivo*, using the mouse skin wound healing model, PEP treatment (3 days) increased MMP activity at the wound edges, along with increased MMP-2 and MMP-9 protein levels, and increased keratinocyte cell proliferation. Altogether, our data suggest PEP stimulates MMP activity, and with a positive effect on early cellular events during wound healing.

## Introduction

Eggshell membrane (ESM) is a promising biological material with a history of use in wound healing. It has been used as an alternative natural bandage on burned and cut skin injuries for more than four hundred years in Asian countries. Recently, a study demonstrated an ESM-positive effect in a rat skin wound model, by decreasing wound closure time [1]. ESM is a highly fibrous material that is readily available as a remnant of the chicken egg processing (breaking) industry, and therefore shows potential as a low-cost ingredient for wound treatment products. However, mechanisms of how ESM actually influences wound healing or the active constituents involved in this process, are far from understood.

The reciprocal actions between cells and their microenvironment form an evolving network during tissue repair, cell proliferation, cell differentiation, migration, and even apoptosis, which are all cellular events that occur during the wound healing process. The extracellular matrix (ECM), a complex network of fibrous proteins, carbohydrates and glycoproteins surrounding the cells, is the main regulator in tissue regeneration. It functions as structural support and scaffold for cell growth and differentiation, supplier and regulator of essential growth factors and cytokines, and cell enzyme activities (for review [2]).

In wound healing, fibroblasts are a key player in maintaining skin homeostasis and for orchestrating physiological tissue repair. A few days after tissue damage, the new stromal tissue is produced. Fibroblasts are recruited from the local connective tissue surrounding wounds, and become highly proliferative to produce ECM for the new granulation tissue accompanied by angiogenesis and the influx of inflammatory cells. In the granulation tissue, the fibroblasts become further activated by differentiation into myofibroblasts with alpha-smooth muscle actin (α-SMA) expression for the contractile properties important for contracting the wound [3]. The final phase of healing consists of a remodeling of the granulation tissue into a more mature and stronger scar tissue. Generation of new stromal tissue is also relevant for the beginning of re-epithelialization and is ensured by local keratinocytes at the wound edges and by epithelial stem cells from sweat glands or hair follicles. Re-epithelialization coincides with the recruitment of dermal fibroblasts, and it is likely that this interaction is important during the rebuilding of tissue integrity [4]. In all these steps, proteolytic enzymes such as matrix metalloproteinases (MMPs) and their inhibitors play a major role. Regulation of fibroblast proliferation and migration occurs through local release of growth factors and cytokines from macrophages or their release from the provisional matrix by MMPs. Remodeling of the ECM into scar tissue is dependent on MMP activity, either directly by degradation of ECM or indirectly by their ability to affect cell behavior [5].

MMPs are classified based on their substrate specificities and include collagenases (MMPs 1, 8, 13), gelatinases (MMPs 2, 9), matrilysins (MMPs 7, 11, 26) and stromelysins (MMPs 3, 10). MMPs are normally secreted as zymogens, which must be processed by proteolytic enzymes intracellularly or extracellularly to generate their active forms or by oxidative cleavage of a “cysteine switch” in their prodomain [6, 7]. More recent studies have demonstrated that MMPs also localize to the nucleus to play distinct roles [8]. Under normal physiological conditions, MMP proteolytic activities are controlled by any of the following three processes: transcription, zymogen activation, and inhibition of the active form by various tissue inhibitors of MMPs (TIMPs) [9]. Certain MMPs such as MMP-2 and MMP-9 are localized to the epithelial-stromal interface behind migrating epithelial cells in wounds [10]. In culture, it has been demonstrated that keratinocytes express both MMP-2 and MMP-9 [11].

The eggshell membranes are a functional equivalent of ECM, which surrounds the egg white and serves both protective and supportive roles during eggshell mineralization [12]. Its composition and structural features have been highly characterized [13]; the meshwork of fibers is approximately 90% proteinaceous [14], and also contains complex carbohydrates such as glycosaminoglycans (GAGs) [15] and N-glycans [16]. Structural proteins such as cysteine-rich eggshell membrane proteins (CREMPS) and collagens (particularly type X) are abundant constituents, together with glycoproteins, Ca-regulatory proteins and enzymes with a disintegrin-like and metalloproteinase domain with thrombospondin type 1 motif (ADAMs) [13, 17–20]. These are all constituents that are relevant from a wound healing perspective [13]. Eggshell membrane extracts have been demonstrated to affect MMP mRNA expression, and to be a positive supplier for the extracellular matrix environment of fibroblasts *in vitro* [21]. In this study, we have studied processed ESM powder (PEP), a material that mimics the structural properties of intact ECM demonstrated in our previous work [16]. Our focus has been on the impact of PEP on fibroblasts and MMP-activity in an *in vitro* cell culture system and an *in vivo* murine wound healing model.

## Materials and methods

### Preparation of eggshell membrane (PEP) powder

Processed eggshell membrane powder (PEP) used in this study was prepared as previously described [16]. PEP was sterilized by gamma irradiation at 25 kGy before *in vitro* (cell culture) and *in* vivo (murine wound model) studies.

### Cell culture and treatment

Human primary dermal fibroblasts (ATCC, Manassas, VA, USA) were cultured in Dulbecco´s modified Eagle´s medium (DMEM) supplemented with 10% fetal bovine serum (FBS), 100 U/ml penicillin, 100 μg/ml streptomycin and 250 μg/ml fungizone (all purchased from Thermo Fisher Scientific, Waltham, Massachusetts, USA) in tissue culture flasks. The cells were maintained at 37°C in a humidified atmosphere of 5% CO_2_ and routinely sub-cultivated twice a week at a concentration of 5.000 cells/cm^2^.

For PEP evaluation experiments, fibroblasts were plated onto 12-well culture plates at a concentration of 150.000 cells/well in medium with 10% FBS and incubated overnight. The cells were then pre-incubated with serum-free medium for 6 hours, before replacing with PEP dissolved in serum-free medium, or with only serum-free medium (control), and further incubated for 3 days. For longer term (10 days) incubation, the fibroblasts were incubated with medium with 2% FBS (control), or PEP or 10 ng/ml transforming growth factor (TGF)-β1 in medium with 2% FBS the first seven days. Thereafter the cells were replaced with serum-free medium (control) or with the different stimulus (PEP or TGF-β1) dissolved in serum-free medium for the last 3 days of the incubation. TGF-β1 was used as a positive control to elicite α-SMA expression [22]. The cells were examined in Leica DM IL LED light microscope (Leica Microsystems, Nussloch GmbH, Germany) during incubation and images were taken by Canon camera EOS 550D (Canon Inc., Tokyo, Japan). The cell medium from 3 and 10 days incubations were collected, centrifuged 5 min at 12,000 rpm to remove cell debris, and then subjected to ELISA and zymography analysis. The cells were washed twice with PBS, lysed in RIPA buffer before subjecting to Western blotting and ELISA analysis. Cells between passages 3–10 were used in experiments in this study.

### Live/dead cell assay

The Live/dead viability/cytotoxicity kit (Molecular Probes, Invitrogen, Paisley, UK) was used according to the manufacturer´s instructions to stain for viable cells in PEP aggregates. This kit is based on the coincident determination of live and dead cells with two probes that distinguish viable from dying cells. In living cells, the nonfluorescent calcein AM is converted to a green-fluorescent calcein after acetoxymethyl ester hydrolysis by intracellular esterases (emits green fluorescence). While in dead cells, ethidium homodimer 1 (EthD 1) bind to DNA and emits red fluorescence.

### Cell proliferation, cell viability and migration assays

Fibroblasts were seeded out onto black (cell proliferation assays) or white opaque (cell viability assays) microtiter plates at a cell density of 3000 cells/well in culture medium containing 10% FBS, and incubated for approximately 24 hours. PEP dissolved in serum-free medium, or only medium (control) was then added to cells and incubated for 3–10 days. Cell proliferation of fibroblasts was measured by using the CyQUANT cell proliferation assay kit (ThermoFisher Scientific), and cell viability was determined with the Cell Titer-Glo Luminescent Cell Viability Assay (Promega, Madison, WI, USA), according to the manufacturer´s instructions. The fluorescence and luminescence intensity of cell proliferation and viability were detected using FLUOstar OPTIMA microplate reader (BMG LABTECH GmbH, Ortenburg, Germany) and Glomax96 Microplate Luminometer (Promega), respectively. Migration measurements were performed by transwells measurements in 24-well cell migrations assay (Cell Biolabs, San Diego, CA) at cell density 1x106 cells/well in serum free culture medium according to the manufacturer´s instructions. Lower chamber contained PEP at a concentration 1 mg/ml.

### Gelatin zymography

MMP activity in conditioned medium of cultured fibroblasts was assessed by gel electrophoresis zymography using Novex 10% zymogram (Gelatin) protein gels (Invitrogen AS, Carlsbad, CA, USA). Equal volumes of samples were mixed with 1% (w/v) SDS sample buffer under non-reducing conditions, and loaded onto the gels. The gels were electrophoresed at 30 mA until 30 min after the bromophenol blue dye had run off the gel. The gels were washed 2 x 15 min in renaturing buffer (50 mM Tris-HCl pH 8.0, 5mM CaCl_2_, 2.5% (v/v) Triton X-100), 1 x 15 min in incubating buffer (50mM Tris-HCl pH 7.5, 5 mM CaCl_2_) at room temperature, and then incubated in incubating buffer for 16 h at 37°C. Gels were stained in staining solution (0.1% Coomassie brilliant blue R-250 (w/v), 50% methanol, 7% acetic acid) for 1 h and destained in 7% (v/v) acetic acid, 20% (v/v) methanol until gelatinolytic activity appeared as a clear band on a blue background. Precision plus protein all blue prestained protein standards (Bio-Rad, Hercules, CA, USA) were also applied on the same gels for assignment of molecular mass. Images of the gels were scanned with an Epson perfection 4990 Photo scanner (Epson America Inc., CA, USA), and files were processed using Adobe Photoshop CS5.1. When necessary, adjustment in brightness and contrast were performed across the entire image.

### ELISA

Levels of MMP-2, TIMP2, and MT1-MMP proteins in the conditioned cell media and cell lysates of cultured fibroblasts were determined using MMP-2, TIMP2 and MT1-MMP DuoSet ELISA kits (R&D Systems, Minneapolis, MN, USA); the levels of shed syndecan-4 were measured using a syndecan-4 ELISA kit (IBL International GmbH, Hamburg, Germany), all according to the manufacturer´s instructions. The optimal sample volumes for each assay was determined before equal volumes of control and PEP treated samples were evaluated with these assays.

### Western blotting

At the end of incubation time, control and PEP or TGF-β1 treated cells were lysed in RIPA buffer (150 mM NaCl, 1% NP40, 0.5% Na-deoxycholate, 0.1% sodium dodecyl sulphate (SDS) in 50mM Tris-HCl, pH 8) with addition of phosphatase inhibitor cocktail and protease inhibitor AEBSF (Sigma-Aldrich), and the cell lysates were homogenized by centrifuging through QIA shredder mini spin columns (Qiagen, Hilden, Germany). Equal volumes of cell lysates were loaded onto 12% Bis-Tris SDS-PAGE gels and electrophoresed for 1 h at 100V, before blotting onto a nitrocellulose membrane using iBlot dry blotting system (Invitrogen, Carlsbad, CA, USA). The membranes were blocked in Tris-buffered saline (TBS)-Tween with 2% ECL primer blocking agent (GE Healthcare, Chicago, USA) for 1 h, incubated with mouse anti-α-SMA polyclonal antibody (ab 5694, Abcam, Cambridge, UK) diluted 1:200 in TBS-Tween with 1% ECL primer blocking agent overnight at 4°C. The membranes were washed 3x 5 min with TBS-Tween, incubated with secondary antibody ECLPlex goat-α-mouse IgG-Cy3 antibody (1:2500; PA43010, GE Healthcare) for 1 h, and then washed 3x 5 min in TBS-Tween. For loading control, the membranes were re-blotted (without stripping) with mouse monoclonal antibody against α-tubulin (1:10.000; T5168, Sigma-Aldrich). The protein bands were visualized using the G:BOX gel documentation system (Syngene, Cambridge, UK) and quantified using ImageJ-win64 software. The expression of α-SMA was presented as the ratio of treatments to the control after normalizing to the loading control tubulin.

### Cell culture immunofluorescence

For fluorescent immunostaining experiments, fibroblasts (10,000 cells/well) were seeded out on Nunc 8-well chamber slides (Thermo Fisher Scientific, MA, USA) and treated with PEP or TGF-β1 as described under cell culture and treatment section. At the end of incubation, cells were fixed in 4% PFA (Reidel-de Haën, Seelze, Germany) for 15min, washed three times in PBS before permeabilizing with 0.5% TritonX-100 in PBS for 15 min. After washing in PBS-tween, the cells were blocked using 5% milk in PBS-tween for 1h before incubation with monoclonal mouse anti-vinculin antibody (1:1000 dilution; V9131, Sigma-Aldrich) for another 1 h. The cells were washed and subsequently incubated with Alexa 546-conjugated goat-anti-mouse secondary antibody (1:400 dilution; Life Technologies, Carlsbad, CA, USA) for 30 min before mounting using Dako fluorescent mounting medium (Glostrup, Denmark). For staining of F-actin filaments, Alexa Fluor 488-conjugated Phalloidin (1:200 dilution; Invitrogen) was added to the cells together with the secondary antibody. The cells were examined by Zeiss Axio Observer Z1 microscope (Zeiss, Jena, Germany), and images were processed using Adobe Photoshop CS5.1. Hoechst dye from Molecular Probes (Invitrogen) was used to counterstain the nuclei. If necessary, adjustment in brightness and contrast were performed manually across the entire image.

Focal adhesion area and circularity were quantified with ImageJ following step by step the protocol described by Horzum et al [23], using 0–50 μm^2^ as cutoff value for FA area. A minimum of 3 images with > 20 cells per image were quantified for each treatment. Actin fibers (the ratio actin fibers at cell border compared with actin fibers intracellularly) were quantified using ImageJ. Presented values are Integrated Densities (sum of pixel values in selection * area of selection). A minimum of 5 images with >10 areas quantified for each treatment. Actin fiber thickness was quantities with ImageJ, following protocol for DiameterJ plugin, with manually masking of the images.

### Mice wound trial

All the *in vivo* experiments were conducted following approval of the animal protocol (CMM-2108) by the University of Ottawa Animal Care Committee and according to the guidelines of the Canadian Council on Animal Care (CCAC). The method of sacrifi was inhalation of carbon dioxide which is approved by the Canadian Council of Animal Care (CCAC) and the Canadian Association for Laboratory Animal Medicine. The activity of MMP-2, MMP-9 and MT1-MMP during wound healing under the effect of PEP was assessed using the mouse excisional wound splinting model [24]. A total of 12 C57BL/6J male mice (10–12 weeks old, weighing 27–30 g) were acclimatized for at least 7 days under ambient conditions (12 hours light/ dark cycle at 22 ± 2°C and humidity 50–60%) with free access to both sterile water and standard rodent soft chow ad libitum. On the day of the experiment (day 0), mice were anesthetized using 5% isoflurane in 100% oxygen (inhalation) and maintain anesthesia using 1–3% isoflurane. The dorsal hair of the mice was removed using an electric razor. The skin was sterilized by chlorhexidine and 70% ethanol washes. Then, a rounded, full-thickness, 6-mm (0.28 cm2) cutaneous wound down to the level of subcutaneous panniculus carnosus was created on each side of the dorsal midline by impressing a biopsy punch instrument on each mouse dorsum followed by grasping and pulling the circular region with a forceps, and excising the full-thickness tissue with scissors. Directly after excising the skin, a silicone splint (6 mm diameter) was centered over each wound area, glued to the skin using super glue and then sutured to the skin with interrupted 5–0 sutures to ensure positioning. After suturing the silicone splint, PEP (2.5 mg) as a suspension in 25 μl of sterile filtered PBS per cm^2^ of wound area was placed into the right wound area, while the left side received an equal volume of PBS alone to serve as a control. The treated and control wound were secured by a commercial dressing (3M Tegaderm hydrocolloid dressing, Minnesota Mining and Manufacturing Company) to prevent wound infection. After surgery mice were placed in individual cages for recovery. At day 3, light anesthesia was induced using 1–3% isoflurane then PEP (as suspension) was re-applied as before, and then wound area was re-covered with Tegaderm. Perioperative antibiosis was achieved with subcutaneous injections of Enrofloxacin (5 mg/Kg) at day 0 and day 1. To reduce pain, buprenorphine HCl (0.05 mg/kg) was administered subcutaneously pre-operatively (day 0), while Carprofen (5 mg/kg) was administered post-operatively (day 0) and on day 1 via subcutaneous injection for post-operative pain relief. Four (4) mice were sacrificed at 3 different time points (days 3, 10, and 17) to identify MMP activities in the different phases of wound healing.

### Histological analysis of mice wounded tissue

Sections (five-μm-thick) of ZBF-fixed [25], paraffin-embedded excisional wound tissue samples from mice were cut on a paraffin microtome (Leica RM 2165, Germany) and mounted on poly-L-lysine-coated glass slides. All histological analyses were carried out on deparaffinized and rehydrated sections: 2 x 5 min in xylene before rehydration in graded alcohol baths and then rinsing with dH_2_O.

#### Hematoxylin & eosin (H&E) staining

Overall wound morphology was revealed by H&E staining. The sections were immersed in H&E staining solution at room temperature for 3 min, rinsed in running water, dehydrated in absolute ethanol and mounted in Eukitt. The sections were then examined with a Leica DM600B microscope (Leica, Germany).

#### In situ zymography

The MMP activities in the wound tissue were assessed using the *in situ* zymography technique. Substrate (200 μl) of dye-quenched (DQ) gelatin (Invitrogen) diluted 1:50 in reaction buffer (50 mM Tris-HCl, 150 mM NaCl, 5 mM CaCl_2_, and 0.2 mM sodium azide, pH 7.6) was added to the tissue sections and incubated in a dark and humid chamber at 37°C for 2 hours. Sections were then rinsed 2 x 5 min in PBS baths, dipped in Milli-Q water and air-dried for a few minutes. The sections were mounted using DAKO Gold anti-fade reagent with DAPI (Invitrogen) and examined with Zeiss Axio Observer Z1 microscope (Zeiss, Jena, Germany). Incubation with only reaction buffer was used as a negative control. Integrated densities at wound edges at Day 3 were quantified using ImageJ. Presented values are Integrated Densities (sum of pixel values in selection * area of selection). Both sides of the wound edge were quantified for each treatment (n = 3 mice).

#### Tissue immunofluorescence

Sections were permeabilized with 0.5% Triton X-100 in PBS for 15 min, and then blocked in 5% non-fat dry milk powder dissolved in PBS. The primary antibodies (MMP2, MMP9, and MT1-MMP; 1:50 dilution) diluted in 5% non-fat dry milk in PBS was incubated overnight at 4°C before washing with PBS for 30 min. Subsequent incubation with secondary antibodies was performed for 2 hours, washing with PBS for 30 min before using Dako fluorescent mounting medium (Glostrup, Denmark). The cells were examined by fluorescence microscopy (Zeiss Axio Observer Z1 microscope), and images were processed using Adobe Photoshop CS3. If necessary, adjustment in brightness and contrast were performed manually across the entire image. Integrated densities at wound edges at Day 3 were quantified using ImageJ for all the antibodies used. Presented values are Integrated Densities (sum of pixel values in selection * area of selection). Both sides of the wound edge were quantified for each treatment (n = 3 mice).

### Statistical analysis

Results were expressed as mean ± standard error mean (SEM) of at least three independent cell seeding experiments performed as technical replicates. Significant variance by treatments in comparison to the control sample was determined either by 1) one-way ANOVA using Dunnett’s multiple comparison test and 2) unpaired two-tailed *t*-test. Differences were considered significant at p<0.05. All statistical analysis was performed in Graph Pad Prism version 7.03 (GraphPadSoftware, La Jolla, CA, USA).

## Results

### PEP increases fibroblast proliferation, syndecan-4 shedding and influences focal adhesion and F-actin organization *in vitro*

**Fig 1:**
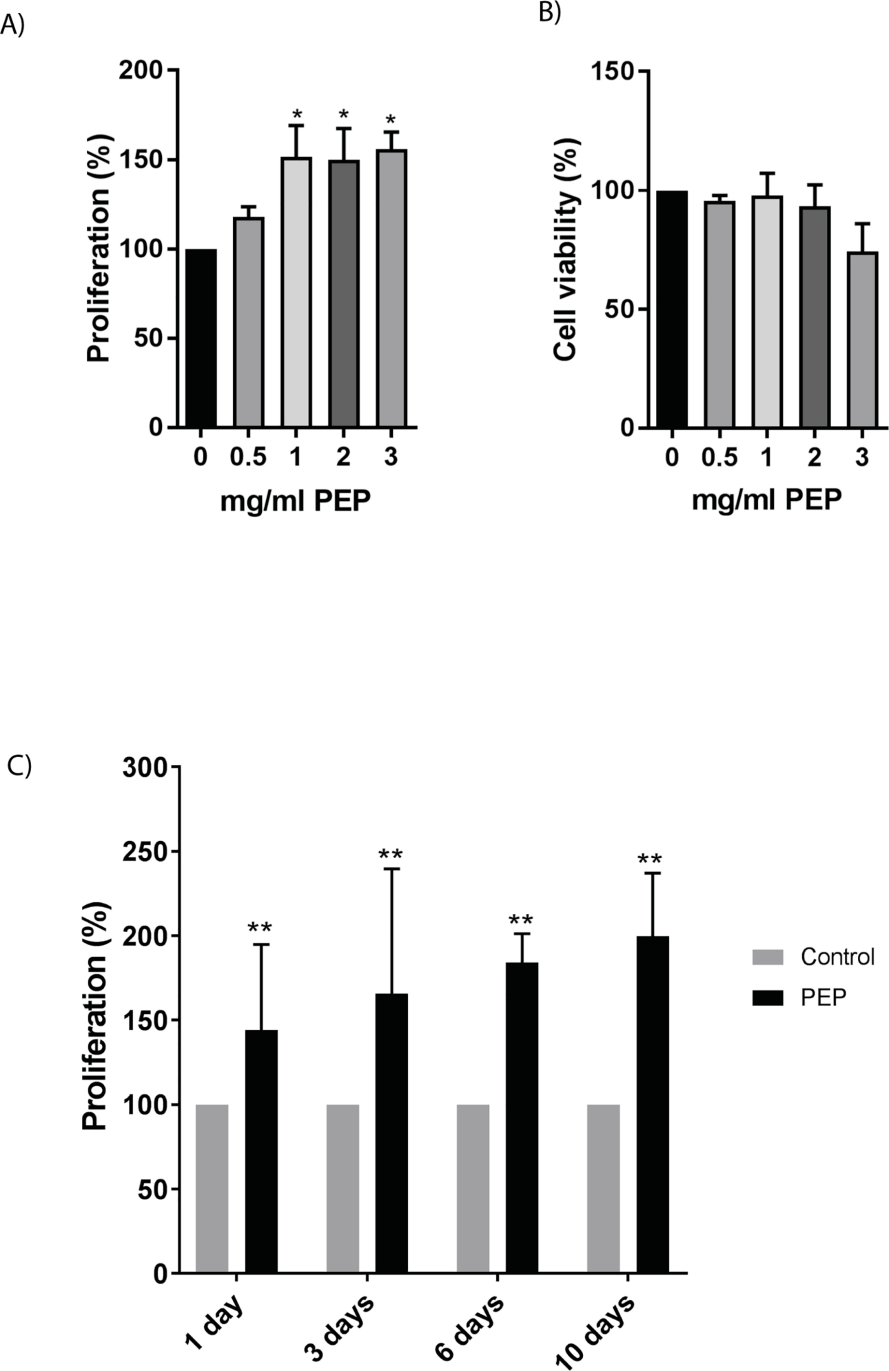
Effect of PEP on fibroblast proliferation and cell viability. Fibroblasts were incubated with different concentrations of PEP (0.5, 1, 2, 3 mg/ml) or left untreated (control). Cell proliferation A) and cell viability B) were assessed after treatment with PEP for 1 day. The data is presented as the average of three independent cell experiments seeded out in triplicates ± SEM. Asterisk indicate significant differences (*p<0.05 in treated cells compared with average mean of control cells for each seeding experiment), statistics assessed by one-way ANOVA with Dunnett’s multiple comparison test. C) Fibroblasts incubated with 1 mg/ml PEP at different time points (1, 3, 6, 10 days). The data is presented as the average of three independent cell experiments seeded out in triplicates ± SEM. Asterisks indicate significant differences (**p<0.01 by unpaired two-tailed t-test in treated cells normalized with control cells at respective time points for each seeding experiment).

Fibroblasts were cultured with and without PEP at different concentrations for various time points and analyzed by CyQuant cell proliferation and cell viability assay (Fig 1). Time points selected were chosen to best mimic *in vivo* mice trials also performed in our study. Day 1–3 represent early wound healing without wound closure, and day 10 represents granulation tissue formation and wound closure. A significant increase in proliferation at 1 mg/ml was obtained after 1 day with no effect on cell viability (Fig 1A and 1B). This concentration was therefore chosen in the following experiments. 1 mg/ml of PEP stimulated cell proliferation already at Day 1, and had a stimulatory effect at all timepoints tested (Fig 1C). Lower concentrations of PEP also stimulated cell proliferation, however at later timepoints (S1 Fig), while higher concentrations led to reduced viability. 1 mg/ml PEP was therefore chosen for the following experiments.

**Fig 2:**
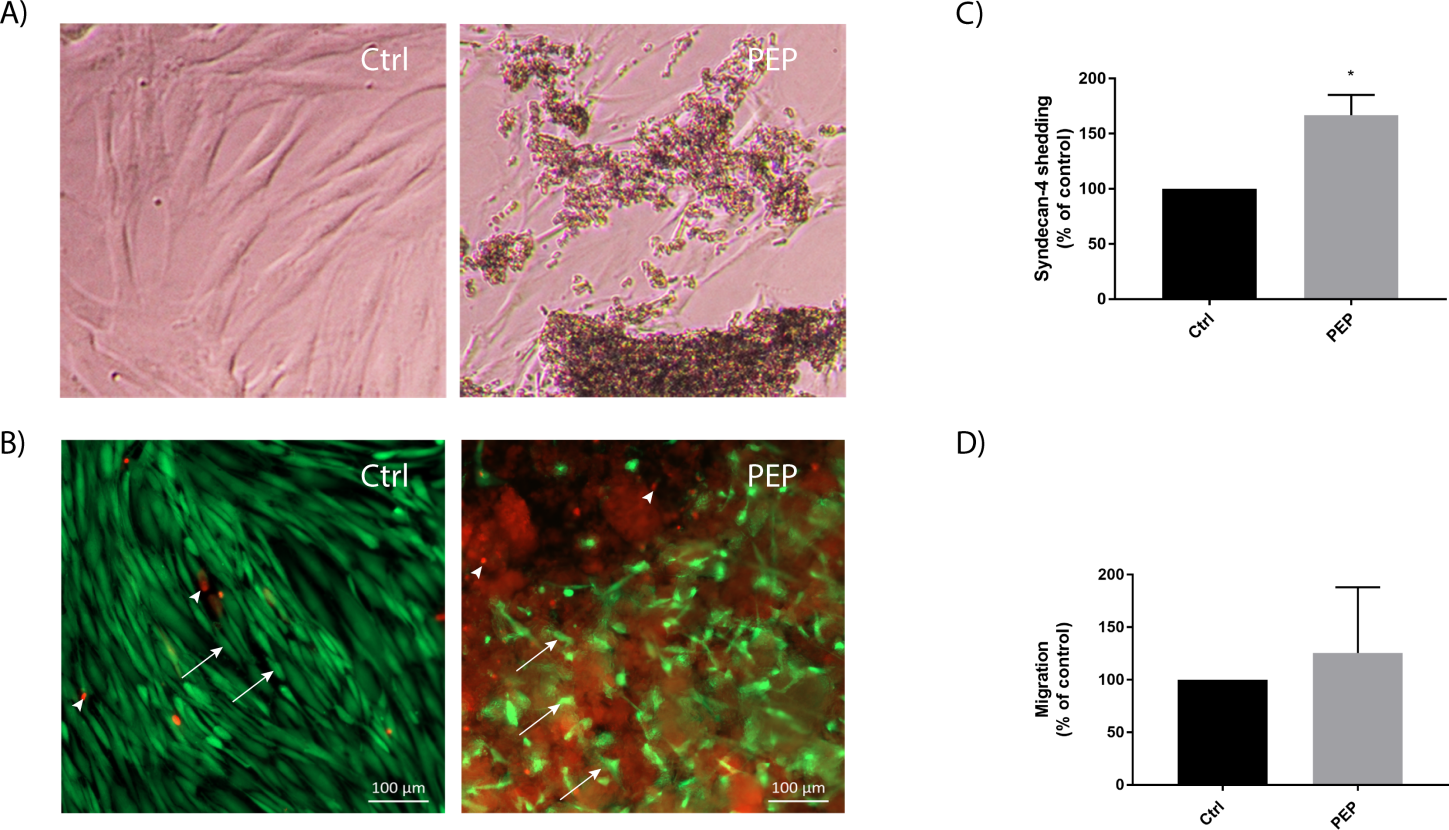
Fibroblasts migrate into PEP aggregates in culture. Fibroblasts were incubated with PEP (1 mg/ml) or left untreated (control) for 3 days, and the cell cultures were then examined by microscopy. A) Light microscopy images showing that fibroblasts are attracted and migrate into PEP aggregates. B) Live/dead cell assay staining showing mainly viable cells (green, arrows) and a few dead cells (red, arrowhead) present in PEP aggregates (diffuse red background). C) ELISA analysis of cell media collected after 3 days incubation with PEP for determination of shed syndecan 4 levels. Data are presented as mean ± SEM from four independent cell experiments in duplicates. Asterisk indicate significant differences (*p<0.05 by unpaired *t*-test in treated cells normalized with control cells for each seeding experiment.). D) Transwell assay showing no effects of PEP as a chemoattractant. Data are presented as mean ± SEM from five independent cell experiments seeded in duplicates.

Light microscopic investigation of fibroblasts cultured with PEP for 3 days (mimicking the same time point as the *in vivo* study) revealed fibroblasts with altered morphology and migrating towards clusters of PEP aggregates (Fig 2A). Live/dead cell assay and fluorescent microscopic analysis revealed mainly viable cells, with only a few dead cells present in the PEP aggregates (Fig 2B). Scratch wound healing assay was performed to try to measure migration *in vitro*. However, since the fibroblasts migrated towards and into the PEP aggregates (as can be seen in Fig 2A), it was technically challenging to measure the directional migration of fibroblasts using this method. We therefore measured other factors important for fibroblast migration. The cell surface protein syndecan-4 is important for cell adhesion by connceting cells to the extracellular matrix[26]. The shedding of the syndecan-4 ectodomain is necessary during cell migration [27]. Our experiments did show a significant increase in shed syndecan-4 protein in the cell media after 3 days of PEP treatment (Fig 2C). Transwells experiments with PEP supplemented in lower chamber was performed to study possible chemoattractant properties of PEP. No significant difference in cell migration was measured by this method indicating PEP without a chemoattractant effect (Fig 2D).

**Fig 3:**
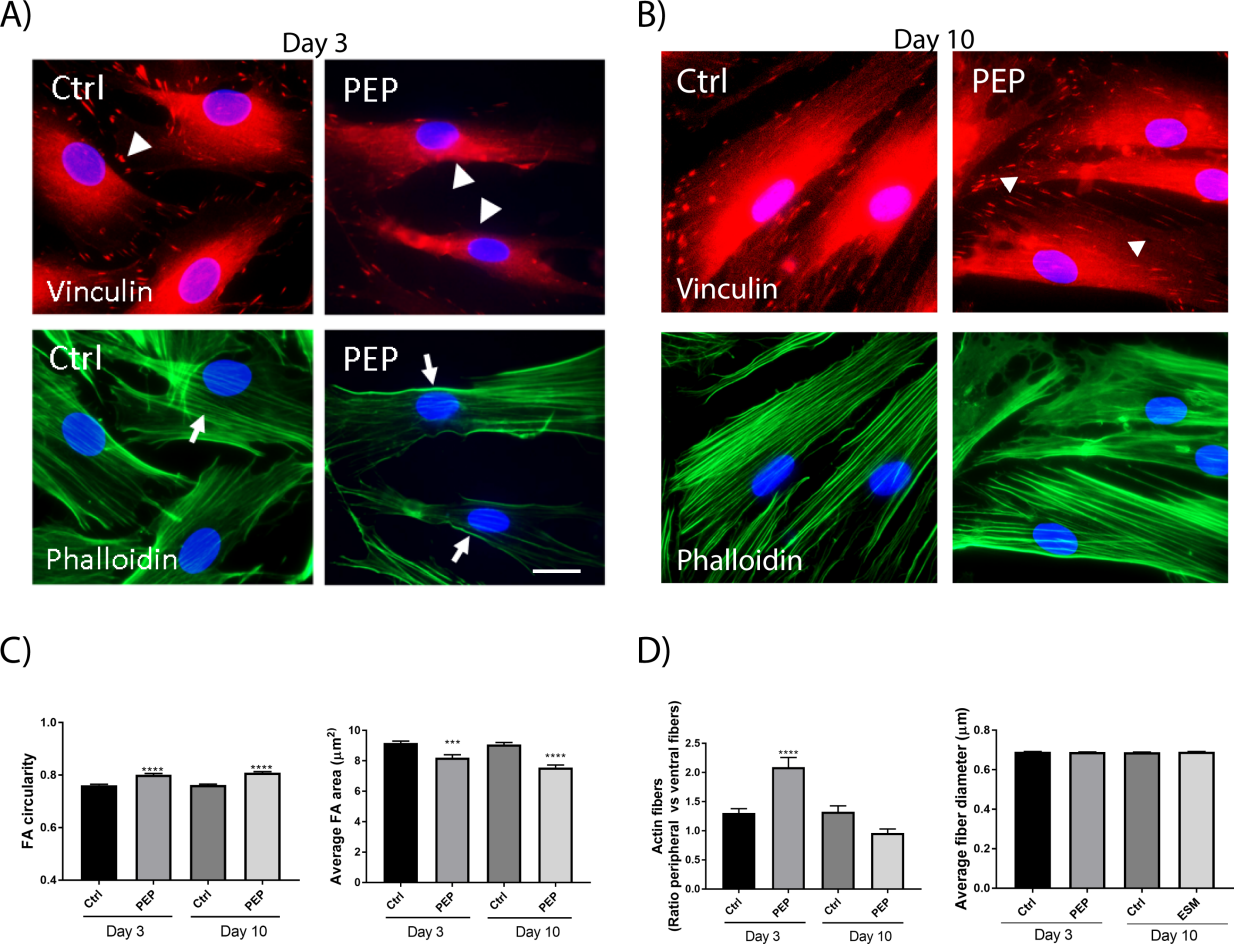
PEP influences vinculin and F-actin stress fibers. Fibroblasts were incubated with PEP (1 mg/ml), or left untreated (control) for 3 days (A) and 10 days (B). Cells were subjected to fluorescent immunostaining for assessing focal adhesion complexes (arrowheads) and F-actin stress fibres (arrows), using vinculin antibody and Alexa 488-phalloidin, respectively. The cell nuclei are stained with Hoechst (blue). C) A reduction of vinculin-positive FAs localized at the cell periphery was observed with PEP, compared to control with clearly visible vinculin-positive FAs at the cell periphery at Day 3. FA area and FA circularity were quantified using ImageJ, based on images of immunostaining, as presented in A and B. A minimum of 3 images with > 20 cells per image were quantified for each treatment, demonstrating that PEP treatment decreased FA area and increased FA circularity at both timepoints. Asterisk indicate significant differences (***p<0.001, **** p<0.0001 in PEP treated cells compared with control cells), statistics assessed by one-way ANOVA with Dunnett’s multiple comparison test. D) The actin filaments were also less prominent intracellularly with a strong staining at the cell borders in these cells compared to control. Actin fibers (the ratio peripheral actin fibers at cell border compared with ventral actin fibers intracellularly, left) were quantified using ImageJ. Presented values are Integrated Densities (sum of pixel values in selection * area of selection). A minimum of 5 images with >10 areas quantified for each treatment. Actin fiber thickness (right) was quantified using ImageJ plugin DiameterJ, quantifying all fibers in the image (n = 5 images per treatment). At Day 10 no difference between control and PEP treated cells were observed. Asterisk indicate significant differences (**** p<0.0001 in treated cells compared with control cells) statistics assessed by one-way ANOVA with Dunnett’s multiple comparison test.

We also investigated the effect of PEP on the organization of focal adhesions (FA), using immunofluorescent staining for the well-used FA marker vinculin and cytoskeletal F-actin filaments. These markers are affected by cell migration and are commonly used for this purpose [28]. After 3 days of incubation with PEP, we observed a reduction of vinculin-positive FAs localized at the cell periphery, compared to control cells. This difference was also evident at Day10 (Fig 3A and 3B upper panels). The FA area was decreased, while the circularity was increased (Fig 3C). The actin filaments were also less prominent intracellularly with a strong staining of peripheral stress fibers at Day 3 upon PEP treatment (Fig 3A lower panels and 3D). This difference in actin filaments was however not evident at Day10 (Fig 3B lower panel and 3D). We did not observe any difference in average fiber thickness upon PEP treatment (Fig 3D).

### PEP stimulates a modest fibroblast differentiation

**Fig 4:**
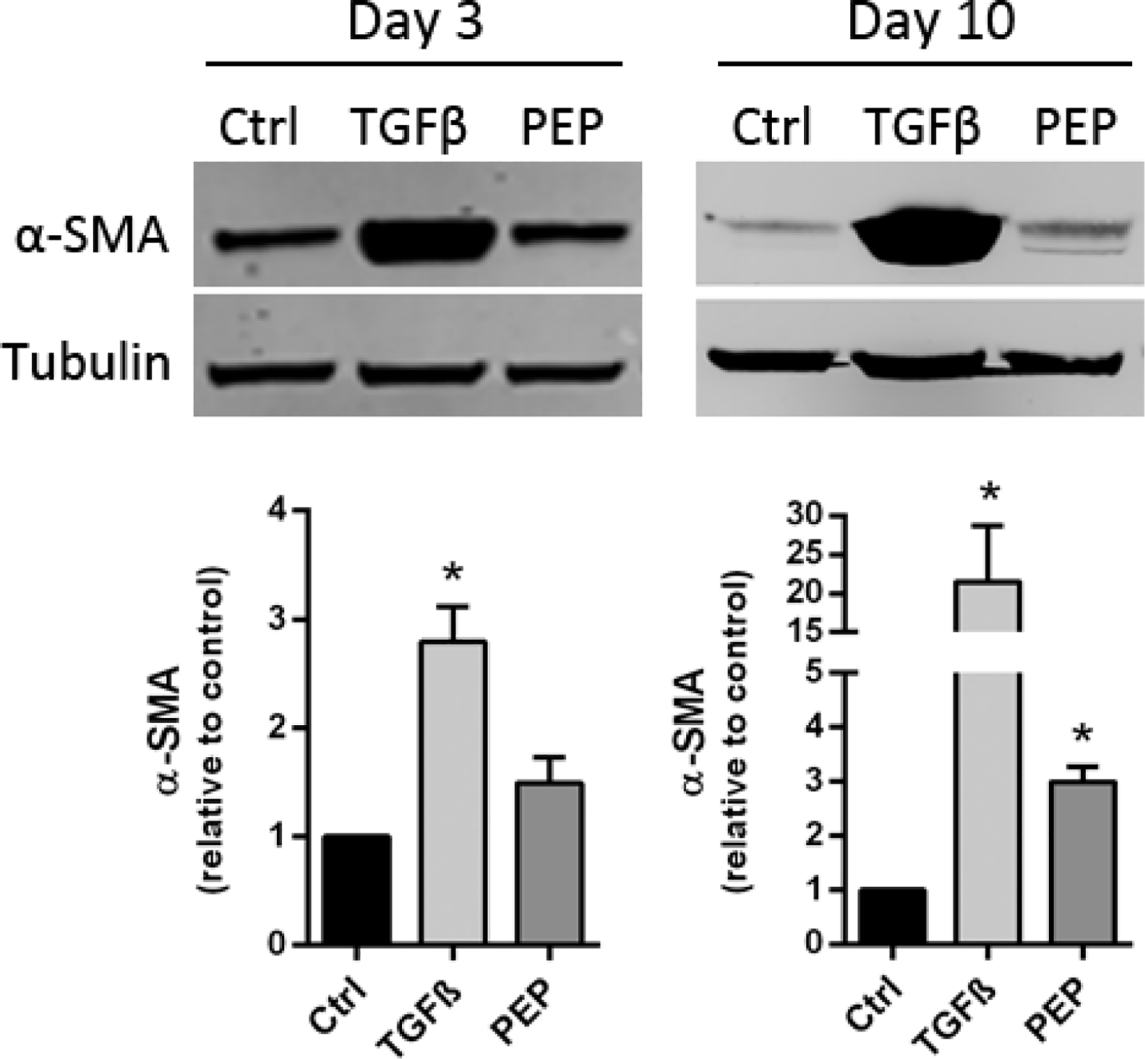
Effect of PEP on myofibroblast differentiation. Fibroblasts were incubated with PEP (1 mg/ml) or left untreated (control) for 3 and 10 days. TGF-β1 (10 ng/ml) was used as a positive control for myofibroblast differentiation. A) Upper panel: Western blotting analysis of α-SMA expression in cell lysates from day 3 and 10 of incubation with PEP and TGF-β1. One representative blot out of three independent experiments is shown. Tubulin was used as a loading control. Lower panel: Quantification of α-SMA expression using densitometric scanning. A significant but modest increase in α-SMA level was obtained after 10 days of incubation compared to the strong inducer, TGF-β1. Data are presented as mean ± SEM from three independent cell experiments seeded out in triplicates (*p<0.05 by unpaired two-tailed *t*-test in treated cells normalized with control cells for each seeding experiment).

We also investigated the impact of PEP on fibroblast differentiation by culturing fibroblasts with and without PEP for 3 and 10 days, using transforming growth factor- β1 (TGF-β1, 10 ng/ml) as positive control for myofibroblast differentiation. This was previously demonstrated to stimulate α-SMA expression in fibroblast culture, and α-SMA is commonly used as a marker of myofibroblasts [22]. Our results show no significant increase in expression of α-SMA at day 3 compared to control. However, a significant increase in α-SMA level was obtained after 10 days of incubation, suggesting a delayed and minor effect of PEP on fibroblast differentiation compared to the strong α-SMA inducer, TGF-β1 (Fig 4).

### PEP increases proteolytic processing of pro-MMP-2 in fibroblasts *in vitro*

**Fig 5:**
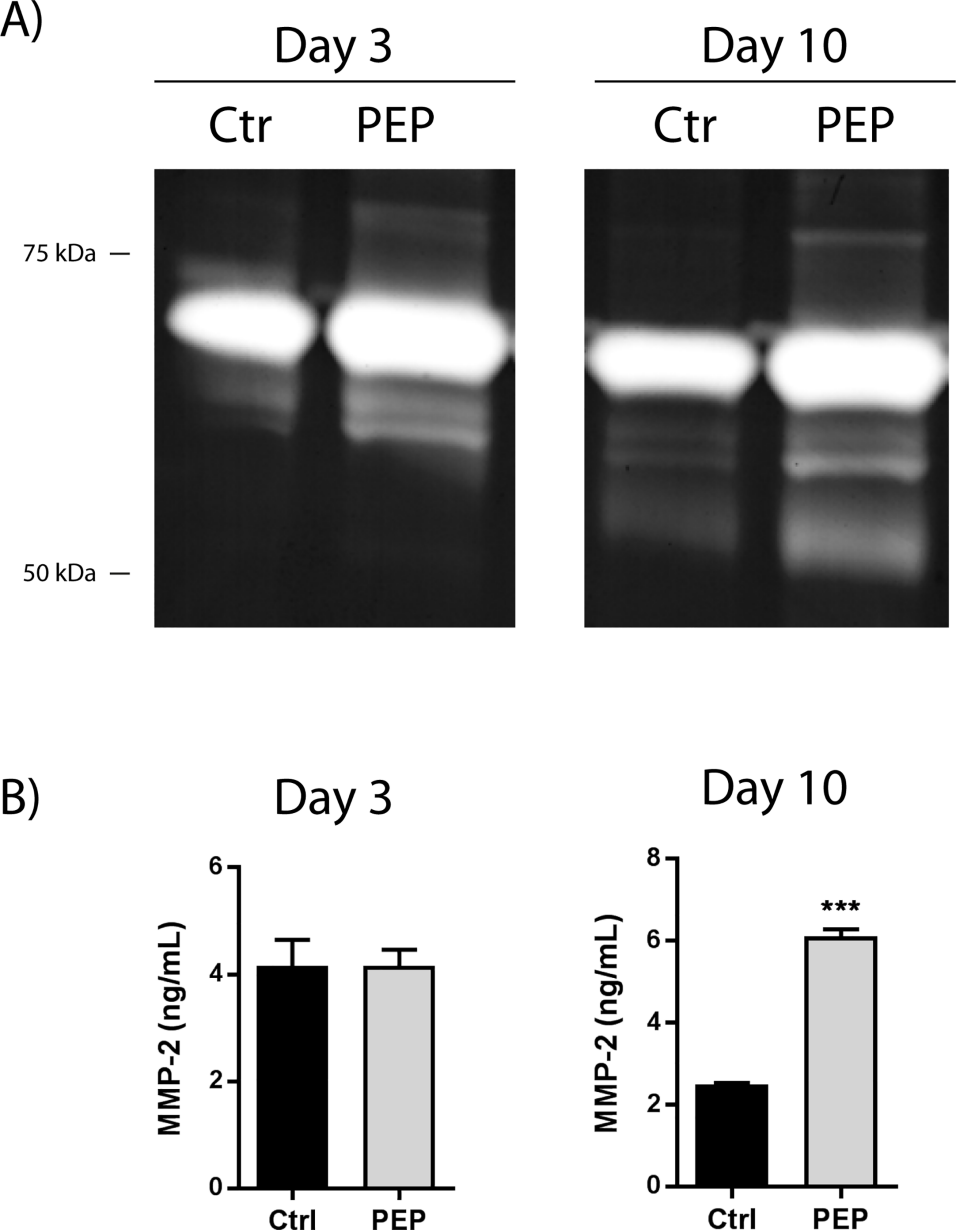
The influence of PEP on MMP-2 processing and MMP-2 levels at short (3 days) and long (10 days) term incubation. Fibroblasts were incubated with PEP (1 mg/ml) or left untreated (control) for 3 and 10 days before the cells were harvested. A) Gelatin zymography analysis of conditioned cell media for assessment of MMP-2 processing. One representative gel out of three independent experiments is shown. B) ELISA analysis for determination of MMP-2 protein levels. Data are presented as mean ± SEM from three independent cell experiments seeded out in triplicates. (***p<0.001 by unpaired two-tailed *t*-test in treated cells compared with control cells).

Since the activities of MMP-2 and MMP-9 are known to be involved in wound healing and the tissue remodeling processes [11, 29], we wanted to investigate whether or not PEP influenced these MMPs in the fibroblast cell culture system. Proteolytic processing of the 72 kDa proMMP-2 to a smaller 62 kDa is known to activate MMP-2 [30, 31]. Activation by phosphorylation, oxidative cleavage or intracellular MMPs was not addressed in this study [32]. Gelatin zymography was used to assess the activation of MMP-2 and MMP-9. A clear appearance of the approximately 62 kDa active form of MMP-2 after 3 and 10 days incubation with PEP was evident compared to the control (Fig 5A). In the longer term (10 days) cultures, there was a further cleavage of MMP-2 as revealed by an additional band of around 50 kDa in the zymogram (Fig 5A). Interestingly, no effect on MMP-2 protein secretion was observed with PEP at day 3. However, after 10 days incubation with PEP, a significant increase of almost 3-fold in MMP-2 secretion was observed (Fig 5B). MMP-9 was not detected in the fibroblast cell cultures, by gel zymography or RT-PCR analysis (data not shown).

**Fig 6:**
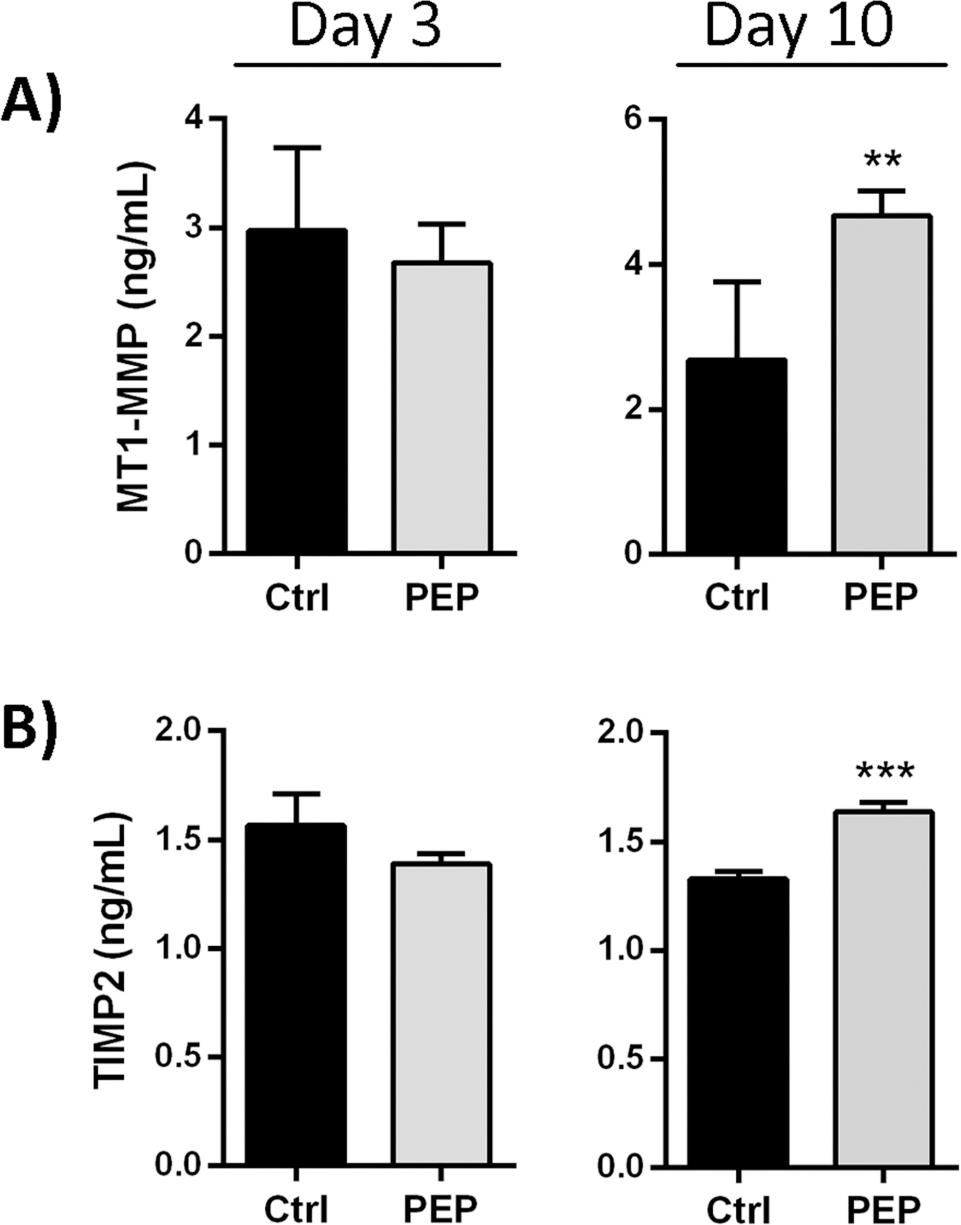
The effect of PEP on MMP-2 regulatory proteins at short (3 days) and long (10 days) term incubation with PEP. Fibroblasts were incubated with PEP (1 mg/ml) or left untreated (control) for 3 and 10 days before cell medium and cell fractions were collected. ELISA analysis of the cell fraction for determining MT1-MMP (A) and the conditioned medium for determining TIMP2 (B) protein levels at day 3 and day 10. Data are presented as mean ± SEM from three independent cell experiments seeded out in triplicates. (**p<0.01 and ***p<0.001 by unpaired two-tailed *t*-test).

MMP-2 activity can be regulated by proteolytic processing or by protein inhibitors, including TIMPs (TIMP-1,-2, 3). In this study, we examined MT1-MMP, the well-known activator of MMP-2, and the inhibitors TIMP-1 and TIMP-2 which inhibit MMP-2 [33]. Fibroblast cultures have been demonstrated to express TIMP-1 and TIMP-2 protein [11]. In our fibroblast culture only TIMP-2 protein was detected, although real-time PCR verified mRNA expression of TIMP-1 (data not shown), reflecting most likely low levels of TIMP-1 protein. In contrast to the stimulation of MMP-2 processing at day 3, PEP did not increase MT1-MMP expression, nor influenced the TIMP-2 expression as compared to the control (Fig 6A and 6B, left panels). Incubation with PEP for 10 days increased the protein expression of MT1-MMP almost 2-fold, while the increase in TIMP-2 levels was more modest, but still significantly elevated (Fig 6A and 6B, right panels).

### PEP stimulates activity and protein expression of MMPs in wound edges *in vivo*

**Fig 7:**
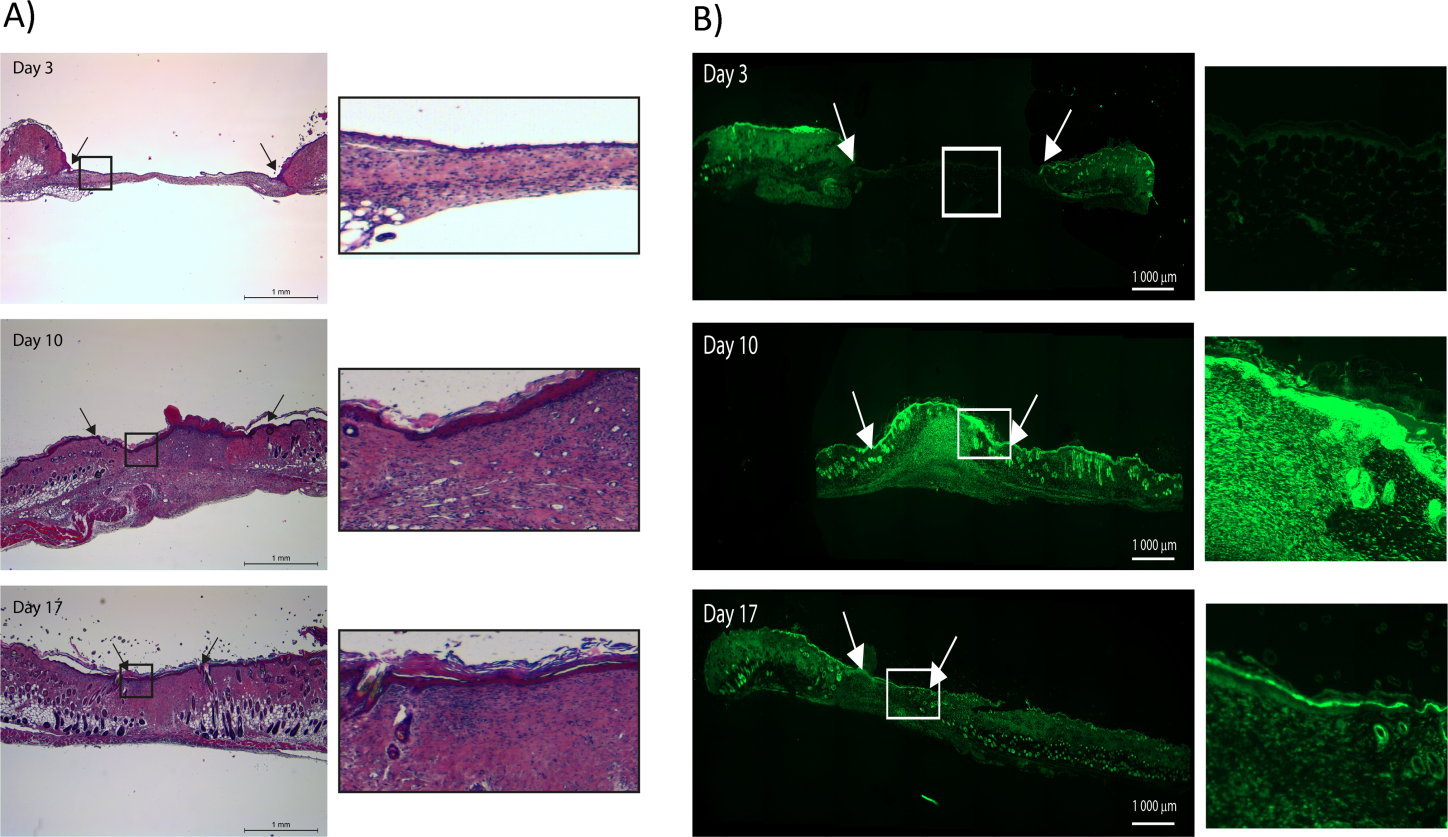
Wound healing in mice models involves MMP activity. A) H&E staining of sections from mice with excisional wounds demonstrate stages of healing at days 3, 10 and 17. B) In situ zymography showing the profile of MMP enzyme activity during the corresponding healing periods. Arrows show the wound edges. Magnification of boxed areas is shown in the right panels in A) and B).

To further understand how PEP affects MMPs during the wound healing process *in vivo*, we investigated MMP activity at different time points during the healing of splinted excisional wounds in mice. Hematoxylin and eosin (H&E) staining of the untreated wound demonstrated normal wound healing phases in the skin; an open wound at day 3, granulation tissue at day 10, and remodeled tissue with a complete epithelial layer at day 17 (Fig 7A). Using *in situ* zymography with DQ-gelatin as a substrate, we demonstrated gelatinase activity in the sections cut from the wound tissues, with an especially high activity localized at the epithelial layer. The enzyme activity was low at the wound edges and almost absent in the wound at day 3. Higher MMP-activity was observed at day 10 in both epithelial layer and the granulation tissue, which then declined in the remodeled tissue by day 17 (Fig 7B).

**Fig 8:**
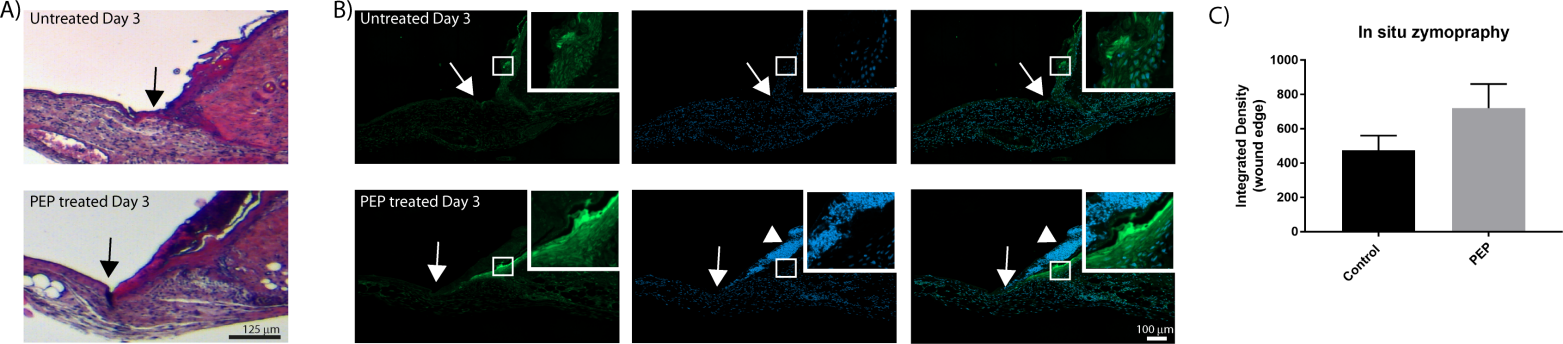
PEP increased MMP activity in the epidermal cell layer at wound edges by day 3 after wounding. A) H&E staining of wound edges at day 3. B) In situ zymography of wound edges at day 3. Green fluorescence indicates the MMP activity. The cell nuclei are counterstained with Hoechst (blue). Arrows show the wound edges. Arrowheads showing increased cell proliferation upon PEP treatment at wound edges. Inserts show magnification of boxed areas, with increased MMP activity. C) Integrated densities of selected for green fluorescence at wound edges at day 3, as presented in inserts in B were quantified using ImageJ. Presented values are Integrated Densities (sum of pixel values in selection * area of selection). Both sides of the wound edge were quantified for each treatment (n = 3 mice).

Gelatinase activity in the healing stages at day 3, was further compared in PEP-treated and non-treated wounds. Higher enzyme activity was observed in the wound edges of PEP-treated wounds at day 3, with a strong staining in the basal membrane beneath the epithelial cells visually. In addition, nuclear staining with Hoechst clearly visualized increased numbers of keratinocytes in this area (Fig 8).

**Fig 9:**
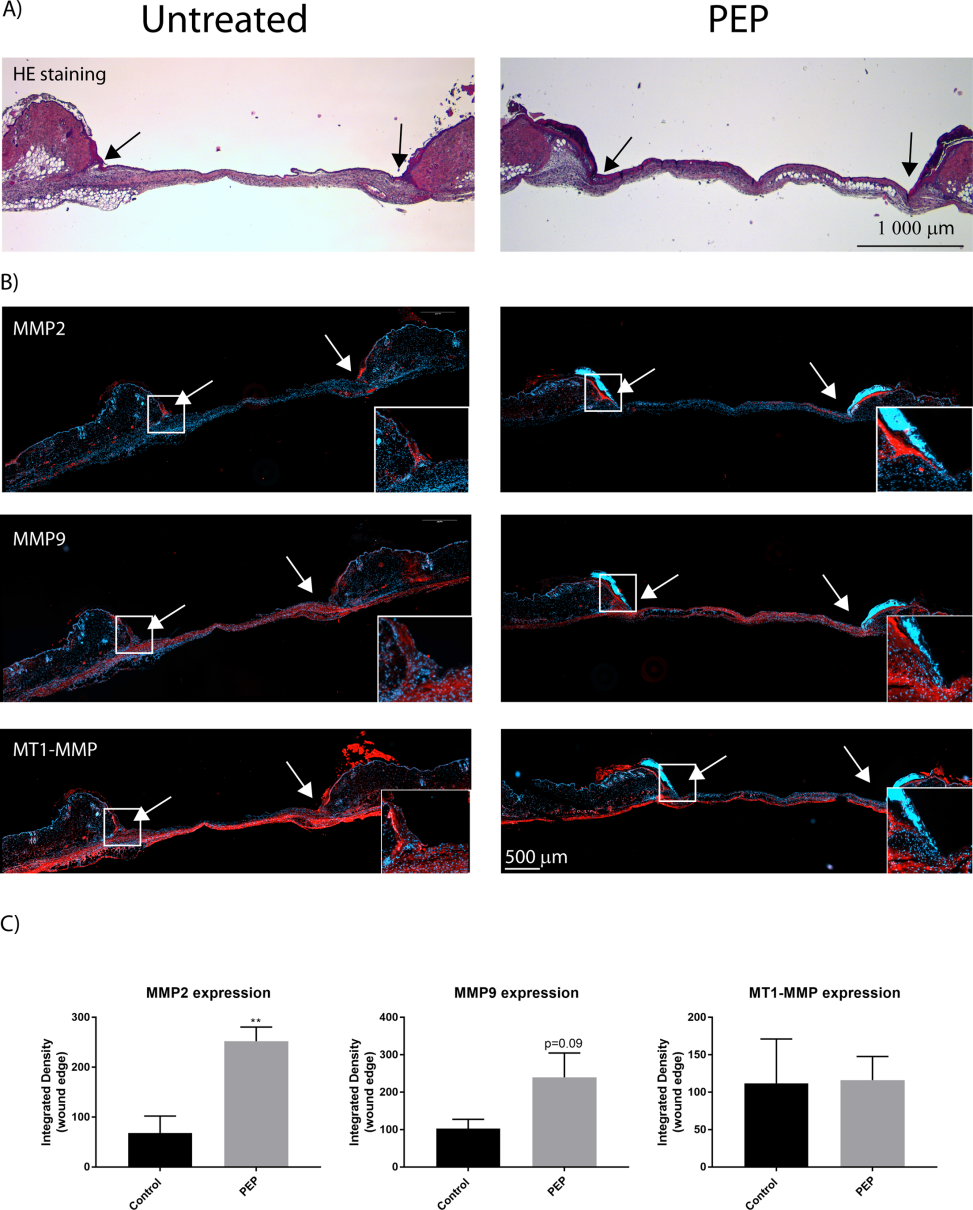
MMP-2 and MMP-9, but not MT1-MMP protein expression are elevated at wound edges with PEP treatment by day 3. A) H&E staining showing the contour of excisional wound sections at day 3. B) Immunofluorescence of excisional wound sections at day 3 with antibodies targeting MMP-2, MMP-9, and MT1-MMP (red). The cell nuclei are counterstained with Hoechst (blue). Arrows show the wound edges. Inserts show magnification of boxed areas for the wound edges. C) Integrated densities of selected areas at wound edges at day 3, as presented in inserts in B were quantified using ImageJ. Presented values are Integrated Densities (sum of pixel values in selection * area of selection). Both sides of the wound edge were quantified for each treatment (n = 3 mice). Asterisk indicate significant differences (**p<0.01 in PEP treated mice compared with control mice) statistics assessed by unpaired two-tailed *t*-test.

Immunostaining with antibodies targeting MMP-2, MMP-9 and MT1-MMP revealed higher protein expressions of MMP-2 and MMP-9 also in this area, while there were no change in the expression of MT1-MMP (Fig 9) in the wound edge area. Following wound healing at day 10, high gelatinase activity was obtained in granulation tissue for both PEP-treated and control wounds. Differences between PEP-treated and control groups at Day 10 and 17 were not significant, due to variability within each group.

## Discussion

Wound healing is an interactive process, relying on the coordination between extracellular matrix components, different cell types and their soluble mediators. Stimulators of this process are highly desirable as ingredients in wound healing products. Processed eggshell membrane powder (PEP) is a biomaterial which is intended for therapeutic use for this purpose. We have recently demonstrated that PEP possesses anti-inflammatory properties with immune cells [16]. Further, *in vivo* data has demonstrated promotion of early wound closure by PEP at day 3 and 10, but not day 17, tested in an excisional wound splinting model in mice (Ahmed et al., submitted). In the present study, we demonstrate for the first time that PEP stimulates cell proliferation and myofibroblast differentiation, and possesses MMP regulatory properties, on fibroblasts *in vitro* and on epidermal cells *in vivo*, suggesting that a stimulating effect due to this biomaterial on MMP-activity is related to fibroblast functionality and re-epithelialization.

Fibroblasts are an important cell type in wound healing with their unique capability to produce matrix components which are necessary for wound closure and replacement of the injured matrix, as well as to induce contraction. Fibroblasts are normally quiescent, but after tissue injury, they become activated to proliferate in the peri-wound stroma and to transmigrate from the collagen-rich connective tissue into the wound provisional matrix of fibrin and fibronectin [34]. Fibroblast proliferation and expansion, migration and myofibroblast differentiation are critical for granulation tissue formation and subsequent healing, and membrane-bound molecules, such as integrin and syndecan-4, are required for this invasive migration [35]. After incubating fibroblasts with PEP for 3 days, fibroblast proliferation was increased compared to control cells, with no effect on cell viability, indicating that PEP is both biocompatible and is a stimulatory material for fibroblasts. The stimulatory effect sustained after longer time incubation. We carried out cell experiments in the absence of serum to reveal the specific effect of PEP, and to avoid the presence of unidentified growth factors and other proteins in the serum. It has previously been demonstrated by others that human dermal fibroblasts can be cultured without serum for longer time periods [36]. They showed that cultured on tissue culture plastic in medium with or without serum for 5 days retained typical spindle-like morphologies.

Incubation with PEP stimulated fibroblast migration towards and into clusters of PEP (day 3, Fig 2). Coinciding with a less adhesive and more migratory phenotype of fibroblasts, a reduction in vinculin area and a redistribution of actin stress fibers were observed at day 3. A similar morphological change for vinculin localization and stress fibers were demonstrated in CD44-deficient (CD44KO) fibroblasts and in vinculin-suppressed 3T3 cells, where the migratory velocity was found to be higher than in the wild-type cells [37, 38]. However, due to difficulties in measuring directional migration in our system using the common scratch wound healing assay, our microscopy data support only indirectly a migratory effect of PEP on fibroblasts (Figs 2 and 3). Our transwell experiments indicate that PEP is not a chemoattractant and the cells have to be in proximity or direct contact with PEP aggregates in order to become migratory. The transition from nonmotile to motile cells is determined by soluble growth factors or an extracellular matrix [39]. PEP contains collagen, fibronectin, fibrin and vitronectin [13] that are reported ECMs that play essential roles in wound healing. It has been demonstrated that collagen matrix initiates dermal fibroblasts motility in the absence of any growth factors, but that platelet-derived growth factor (PDGF) do not stimulate migration alone without a collagen matrix [40]. We observed increased shedding of Syndecan-4 after treatment with PEP compared to control, indicative of a direct or indirect role of PEP on this cell surface membrane molecule. Syndecan-4 is important for the assembly of FA, cell attachment and spreading [41]. It has also been suggested to be a mechanosensing molecule in the plasma membrane [42]. Cleavage of Syndecan-4 ectodomain results in an altered distribution of cytoskeletal components, functional loss of adhesion, and gain of migratory capacities [27]. The α5β1-integrin provides a mechanical connection between the fibroblast and fibronectin and is necessary for migration of fibroblasts toward fibronectin-rich extracellular matrix in the wound [43]. However, Syndecan-4 is necessary for a complete adhesion-dependent signaling response and acts as the initial fibronectin sensor [44, 45]. Both MMP-2 and MMP-9 can cleave syndecan-4[46]. We have demonstrated that PEP activates MMP-2 in fibroblasts at day 3 (Fig 5), and therefore believe this active MMP-2 to shed syndecan-4 detected at this time point. However, ADAMs (disintegrin and metalloprotease), other metalloproteinases [47], and many other serine proteases[48] have also been demonstrated to cleave Syndecan-4. Interestingly, proteomic data from our group demonstrated that PEP contains ADAMs[13]. Proteomic studies of eggshellmembrane or PEP did not detect the presence of various MMPs using MS-based proteomics [13, 19, 49]. However, tissue inhibitor of metalloproteinase 3 (TIMP3) has been identified in both PEP [13] and ESM proteome [13, 19] At present it is not known if PEP directly stimulates shedding by proteases present, or indirectly by another mechanisms stimulating the observed MMP-2 activation.

Our *in vitro* data demonstrated that PEP after longer time incubation (day 10) increased the level of the contractile protein and myofibroblast marker, α-SMA, thus indicative for a stimulation of fibroblast-to-myofibroblast differentiation. Myofibroblasts in granulation tissue exert tension on the ECM and contract the matrix, bringing the wound margins closer together, which promotes wound closure [50, 51].The effect of PEP seems to be a long-term process as α-SMA levels were only weakly significantly increased after day 10, in contrast to the positive impact of the myofibroblast stimulator TGF-β1, which showed significantly increased α-SMA levels already at day 3. Bundles of contractile microfilaments and supermature FAs are two of the major ultrastructural features that discriminate highly mature myofibroblasts from quiescent fibroblasts [52]. Supermature FA is elongated structures arising from the fusion of several FA and always associated with α-SMA [53]. The formation and stability of supermature FAs were shown to be dependent on the α-SMA-mediated contractile activity of myofibroblast stress fibers [54]. Visual inspection at day 10 revealed some cells with elongated FA (Fig 3). However, quantification of all FA in the cells at day 10 did not reveal a major induction of these elongated structures (supermature FA). We suggest the modest increase in α-SMA at day 10 most likely reflects heterogenity in the cell population, with some cells more differentiated at that time point. We believe that fibroblasts are first attracted to PEP, and migrate towards and into PEP clusters followed by induction of myofibroblasts differentiation at day 10. In our previous publication, SEM picture outlined the PEP aggregates, and their structural properties and “scaffold” properties [16].

We show that PEP stimulates the processing of proMMP-2 to 62 kDa active zymogen at both day 3 and day 10. However, our data revealed that PEP increased the levels of MMP-2, MT1-MMP, and TIMP2 only in the longer time cultures (day 10), reflecting a specific regulatory pattern and/or different regulatory pathways influenced by culture time. Increased MT1-MMP protein levels *in vitro*, together with increased active MMP-2 levels and simultaneous with increased α-SMA expression at day 10, support a possible involvement of MMP activity in myofibroblast differentiation. In addition, we also observed a further cleavage of MMP2 to the lower molecular weight (~50 kDa) active form after treatment with PEP, similar to TGF-β1 treatment, at day 10. Whether this activity is linked to a specific (secondary) cleavage site by MT1-MMP or autolysis on MMP-2, and how it is related to the fibroblast differentiation process was not further studied. The effect of PEP on MMP activation by phosphorylation or oxidative cleavage and intracellular MMPs [32] was not addressed in this study, and therefore other activation mechanisms could be possible.

Regulation of MMP2 activity occurs via different mechanisms, of which regulation through TIMP2 and its cell surface receptor MT1-MMP is finally decisive. One target of MT1-MMP is activation of proMMP-2 and knockdown of MT1-MMP in skin fibroblasts, which results in the loss of ability to activate proMMP-2 [55]. A balanced expression of MMPs and TIMPs is critical for wound repair. TIMP2 at low levels is necessary for recruitment of proMMP-2 to MT1-MMP at the cell surface and subsequent activation. However, at high levels of TIMP2, the activation of MMP-2 by MT1-MMP is inhibited [56]. The involvement of MMPs and their regulators in tissue repair is well known, and their induced expression during normal excisional repair that peaks with the well-characterized inflammatory and granulation stages of repair has been demonstrated [57]. Their dysregulation induces increased scar-tissue formation, delayed wound healing and non-healing chronic diabetic wounds [58]. In human chronic wounds, a decreased level of MMP-2 activity and increased amounts of TIMP-1 and TIMP-2 has been observed [59]. During metastasis, MMPs activities are important for ECM degradation, in addition to release and activation of growth factors necessary for cell proliferation [60–63].

MMP-9 is another gelatinase that has been implicated in various aspects of tissue development, maintenance, and repair [64], and was shown to be expressed in dermal fibroblasts together with MMP-2 and MT1-MMP [65]. However, MMP-9 was not detectable in our fibroblast culture. One explanation can be that our fibroblasts originate from a subpopulation in the dermis which expresses very low MMP-9 levels [66]. Our *in vivo* data visualized expression of MMP-2 and MMP-9 in granulation tissue and epidermal tissue during healing. MMP-2 and MMP-9 have both been detected in dermal fibroblasts and epidermal cells in other studies [67]. MT1-MMP is mainly distributed in the cell membrane and cytoplasm of epithelial cells and fibroblasts in mice skin samples. During healing of our excisional wound mouse model, MMP enzyme activity was particularly strong in the epidermal basal layer at day 3, spreading out into granulation tissue at day 10, which then declined in the remodeling of tissue observed at day 17. A temporal pattern of gelatinase activity expression has also been observed by others; it is high early after wounding and declines with healing to return to baseline levels when epithelialization is complete [68]. In our study, supplementation with PEP significantly increased MMP activity in the epidermal basal layer at the wound edges by day 3 post wounding. MMP-2 and MMP-9 protein expressions also increased in this basal epidermal layer. Altogether our data support a stimulating effect of PEP on MMP activity and their protein expression in the wound edges. Re-epithelialization requires epidermal cells at the wound edges to loosen adhesion to the extracellular matrix, start proliferation and induce migration across the provisional matrix of the wound. Different MMPs such as MMP -1, 3, 9, 10 and 14 are involved in this process [29]. MMP-9 protein expression is first induced with the onset of repair, mainly in keratinocytes, and decreases following re-epithelialization. In contrast, MMP-2 increases after injury and is constitutively expressed during the healing period [68, 69]. Both MMP-2 and MMP-9 have been suggested to play a role in keratinocyte migration *in vitro*, as the rate of re-epithelialization was significantly delayed in MMP-9 KO transgenic mice compared to WT controls and the migration of keratinocytes was suppressed *in vitro* by MMP-9 or MMP-2 inhibition [70–72]. Our study demonstrates an increased cell density of keratinocytes at wound edges with PEP supplementation, reflecting an early stimulating effect, and in line with our *in vivo* data demonstrating promotion of early wound closure by PEP at day 3 (Ahmed et al., submitted). Injection of the vascular endothelial growth factor (VEGF)-E into wounded skin has been observed to elicit increased epidermal thickening and keratinocyte number, as well as increased MMP-9 and MMP-2 expression [73]. Cell surface MT1-MMP plays a role in cell migration as well in invasion [74] and localizes at the migrating front in keratinocytes accompanied by pro-MMP2 activation [73]. In our study, MT1-MMP expression was not increased in the early wound edge by PEP treatment. Also in fibroblast culture, MT1-MMP was not increased after short time in culture but only after long time culture. MT1-MMP expression in keratinocytes is dispensable for skin homeostasis and repair but also plays a crucial role in the epidermal-dermal crosstalk leading to modulation of vessel density [75]. The clinical outcome of this material in treatment of different wound types is at present unknown, but our results suggest this to be effective during wound healing. We have previously demonstrated that this material holds promising anti-inflammatory effects [16] and activating of epithelization and fibroblast in this study could indicate a broad specificity in treatment. Also, Guarderas et al showed that chicken egg-membrane dressing significantly improved healing of cutaneous wounds in early stages of wound healing in Spraque-Dawley rats [1]. Cutaneous wound repair and chronical wounds express specific MMP-profiles [57, 58], and further studies on effects on MMP-activity in different wound models would be necessary.

## Conclusions

During wound healing, it is crucial that keratinocytes and fibroblasts migrate into the wound site and start to proliferate, in order to re-epithelize and reconstitute the various connective tissue components. We demonstrate that PEP, with its fibrous structure and collagen components, enhances fibroblast and keratinocyte proliferation, myofibroblast differentiation, and regulates the activity of various MMPs. PEP can, therefore, be seen as an accelerator in the first phase of wound healing which enables cells to restructure new tissues.

## Supporting information

S1 Fig Effect of PEP on fibroblast proliferation. Fibroblasts incubated with 0.5 mg/ml PEP at different time points (1, 3, 6, 10 days). The data is presented as the average of three independent cell experiments seeded out in triplicates ± SEM. Asterisks indicate significant differences (**p<0.01 by unpaired two-tailed t-test in treated cells normalized with control cells at respective time points for each seeding experiment).
(TIF)
