# Supplementary figures and images for: Processed eggshell membrane powder regulates cellular functions and increase MMP-activity important in early wound healing processes
Source: PLoS One. 2018 Aug 6;13(8):e0201975. doi: 10.1371/journal.pone.0201975 (PMC6078314; doi:10.1371/journal.pone.0201975)

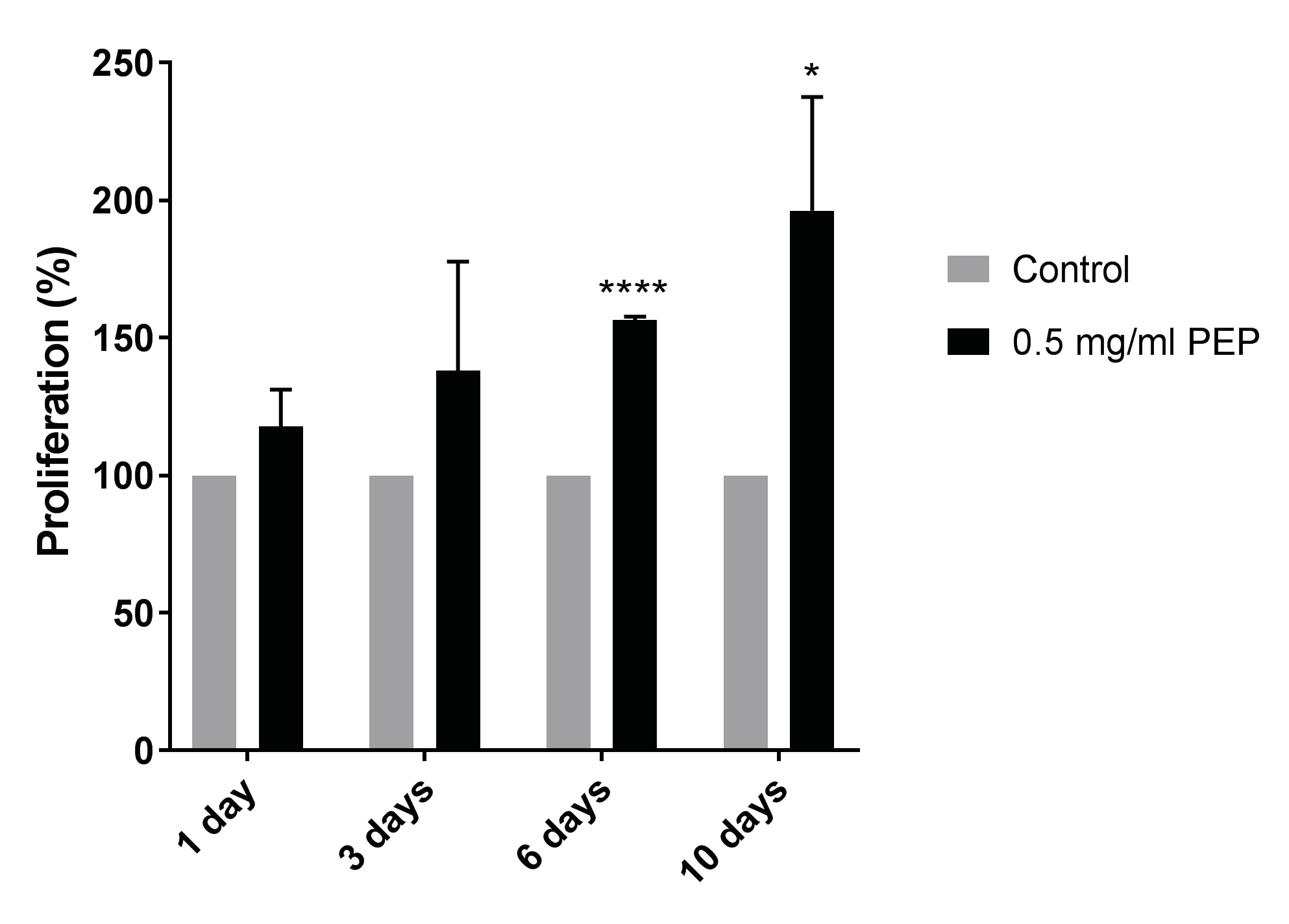

Supplement: S1 Fig — Fibroblasts incubated with 0.5 mg/ml PEP at different time points (1, 3, 6, 10 days). The data is presented as the average of three independent cell experiments seeded out in triplicates ± SEM. Asterisks indicate significant differences (**p<0.01 by unpaired two-tailed t-test in treated cells normalized with control cells at respective time points for each seeding experiment). (TIF) [file pone.0201975.s001.tif]
